# Reversing the pipeline? Implementing public health evidence-based guidance in english local government

**DOI:** 10.1186/s13012-017-0589-5

**Published:** 2017-05-12

**Authors:** Lou Atkins, Michael P. Kelly, Clare Littleford, Gillian Leng, Susan Michie

**Affiliations:** 1 Centre for Behaviour Change, University College London, 1-19 Torrington Place, London, WC1E 7HB UK; 2 Department of Public Health and Primary Care, Institute of Public Health, University of Cambridge, Cambridge, CB2 0SR UK; 3 National Institute for Health and Care Excellence, 10 Spring Gardens, London, SW1A 2BU UK

**Keywords:** Public health, NICE guidelines, Local government, Knowledge transfer

## Abstract

**Background:**

In the UK, responsibility for many public health functions was transferred in 2013 from the National Health Service (NHS) to local government; a very different political context and one without the NHS history of policy and practice being informed by evidence-based guidelines. A problem this move presented was whether evidence-based guidelines would be seen as relevant, useful and implementable within local government. This study investigates three aspects of implementing national evidence-based recommendations for public health within a local government context: influences on implementation, how useful guidelines are perceived to be and whether the linear evidence-guidelines-practice model is considered relevant.

**Methods:**

Thirty-one councillors, public health directors and deputy directors and officers and other local government employees were interviewed about their experiences implementing evidence-based guidelines. Interviews were informed and analysed using a theoretical model of behaviour (COM-B; Capability, Opportunity, Motivation–Behaviour).

**Results:**

Contextual issues such as budget, capacity and political influence were important influences on implementation. Guidelines were perceived to be of limited use, with concerns expressed about recommendations being presented in the abstract, lacking specificity and not addressing the complexity of situations or local variations. Local evidence was seen as the best starting point, rather than evidence-based guidance produced by the traditional linear ‘evidence–guidelines–practice’ model. Local evidence was used to not only provide context for recommendations but also replace recommendations when they conflicted with local evidence.

**Conclusions:**

Local government users do not necessarily consider national guidelines to be fit for purpose at local level, with the consequence that local evidence tends to trump evidence-based guidelines. There is thus a tension between the traditional model of guideline development and the needs of public health decision-makers and practitioners working in local government. This tension needs to be addressed to facilitate implementation. One way this might be achieved, and participants supported this approach, would be to reverse or re-engineer the traditional pipeline of guideline development by starting with local need and examples of effective local practice rather than starting with evidence of effectiveness synthesised from the international scientific literature. Alternatively, and perhaps in addition, training about the relevance of research evidence should become a routine for local government staff and councillors.

**Electronic supplementary material:**

The online version of this article (doi:10.1186/s13012-017-0589-5) contains supplementary material, which is available to authorized users.

## Background

Evidence-based guidelines are produced worldwide to inform clinical and public health practice; for example, in the UK by the National Institute for Health and Care Excellence (NICE); in the US by the National Guideline Clearinghouse and U.S. Preventive Services Task Force; in Canada by the Canadian Task Force on Preventive Health Care and in Australia by the National Health and Medical Research Council. Despite the considerable financial and human investment into the development and dissemination of guidelines, suboptimal implementation of recommendations within guidelines is documented in a range of arenas including public health [1–3]. Barriers to implementing evidence-based recommendations include practical factors such as limited time and resources, and motivational ones such as seeing evidence as having limited relevance and capability ones such as guidelines being difficult to interpret [4].

In England, in 2013, responsibility for the delivery and commissioning of many public health services transferred from the National Health Service (NHS) to local government, the largest reorganization of public health since the creation of the NHS in 1948 [5]. Mandatory services included providing access to sexual health services, protecting population health such as through immunisation and screening programmes, providing NHS commissioners with public health advice, National Child Measurement Programme (NCMP) and NHS Health Check assessment. Knowledge translation, that is, strategies to optimise implementation of the extant public health evidence base and the guidelines arising from them, are needed to ensure that evidence-based recommendations for public health are implemented in the new environment of local government. The research reported in this paper contributes to the development of such knowledge translation strategies.

NICE has been developing public health guidelines since 2005. Although broad in scope and reach, the principal audiences for the guidelines were originally the public health teams working in the NHS who were responsible for implementing guidelines in the context of local variation. The NHS was by 2005 already well used to receiving and implementing NICE Technology Appraisals and Clinical Guidelines. The way that public health guidelines were produced by NICE drew heavily on the methods of evidence-based medicine, an approach which was widely understood if not always supported, in the NHS [6, 7]. This study focusses on the way ideas derived from evidence-based medicine fared in the very different environment of local government after 2013 where the NICE Public Health Guidelines found themselves vying for attention amidst a greater plurality of ways to make decisions than had been the case in the NHS such as, for example, political influences. Until 2013, local government had had neither necessary link nor obligation to comply with NICE guidance. As the public health system has now been reorganised, a scientific understanding of the system changes, what will be required to meet the needs of the system and how best to engage with it, will inform how those who produce and those who implement evidence-based-guidance adapt. This research on evidence-based guidelines for public health focusses primarily but not exclusively on guidance produced by NICE. The research questions this study sought to answer were:

- What are the influences on implementation of guidelines?
- How useful are guidelines perceived to be?
- To what extent is the linear evidence-guidelines-practice model fit for purpose?

## Methods

### Participants and recruitment

Thirty-one interviews were conducted in four local government with councillors, local government officers (Directors of Public Health, officers working directly on public health issues, officers based in other departments whose work related to public health issues, and officers based in a district council in a two-tier authority) and with members of Clinical Commissioning Groups. Potential case study sites were identified through contacts already known to research team members, through a short article about the research project circulated in a newsletter produced by the Association of Directors of Public Health, and through approaches made to attendees at a NICE local government event. The final four case study sites were selected to reflect a range of local government characteristics:

- A unitary authority in the north of England
- An outer London borough
- A two-tier authority with rural areas
- An inner London borough

Table 1 presents a breakdown of the roles of interviewees in each local government.

**Table 1:** Participant role in each local government site

Role	Site 1	Site 2	Site 3	Site 4
Director of Public Health Public health team Clinical Commissioning Group Other LA departments District council Councillor	1 2 1 4 0 2	1 2 1 2 0 1	1 2 2 0 2 2	1 3 0 0 0 1
Total	10	7	9	5

### Procedure

#### Interview schedule development

An interview schedule was developed covering topics relevant to the research questions and using a theoretical framework that focussed on the behaviour change required to increase implementation of evidence. The theoretical framework has three broad elements required for behaviour change: capability, opportunity and motivation (COM-B model)[8] and has been used to understand implementation problems with public health guidance [9, 10]. Each component of COM-B is further divided into two: capability into physical and psychological, motivation into reflective and automatic, and opportunity into physical and social. Following pilot interviews in a separate local authority and examination of transcripts of these interviews, the schedule was revised. The interview schedule is appended (Appendix).

#### Data collection and transcription

Interviews were conducted face to face and audio-recorded, with the recordings subsequently anonymised and transcribed by a UCL-approved professional transcribing service. Interview subjects were sent the transcription of their interview and given an opportunity to correct errors or to clarify anything they felt was unclear. Local government officers were asked about their job role prior to 2013, to identify whether they came from an NHS or a local government background. Table 2 presents a breakdown for each interview transcript of the interviewee’s current job role, their role prior to 2013, and the wave of analysis in which the transcript was included. Of the 21 officers, only a third were working in a local government prior to 2013.

**Table 2:** Participant current and previous role

Participant ID	Role	Pre-2013 role	Wave
1 Off 1	Director of Public Health	Other health	2
1 Off 2 1 Off 3	Public health team Public health team	NHS NHS	2 3
1 Off 4	Clinical Commissioning Group	–	3
1 Off 5 1 Off 6 1 Off 7 1 Off 8	Other LA departments Other LA departments Other LA departments Other LA departments	Local government Local government Local government Local government	2 3 3 3
1 Cllr 1 1 Cllr 2	Councillor Councillor	– –	2 2
2 Off 1	Director of Public Health	NHS	1
2 Off 3 2 Off 4	Public Health team Public Health team	NHS NHS	3 1
2 Off 5	Clinical Commissioning Group	–	2
2 Off 6 2 Off 7	Other LA departments Other LA departments	Local government NHS	2 3
2 Cllr 2	Councillor	–	1
3 Off 1	Director of Public Health	Other	1
3 Off 2 3 Off 3	Public health team Public health team	NHS NHS	1 1
3 Off 4 3 Off 5	District council District council	Other Local government	2 3
3 Off 6 3 Off 7	Clinical Commissioning Group Clinical Commissioning Group	– –	2 3
3 Cllr 1 3 Cllr 2	Councillor Councillor	– –	3 2
4 Off 1	Director of Public Health	Other	1
4 Off 2 4 Off 3 4 Off 4	Public health team Public health team Public health team	Local government NHS Other health	1 1 1
4 Cllr 1	Councillor	–	1

### Data analysis

Analysis of the 31 interview transcripts was conducted in three waves, with 11 transcripts analysed in the first wave and 10 analysed in each of the second and third waves. Conducting the analysis in three waves allowed the researchers to develop, refine and apply an analytical framework, including identifying themes that emerged from the data during analysis. Examining the transcripts in three waves allowed a process of triangulation within the data, confirming which findings could be generalised across all four case study sites. Examination of the third wave of transcripts confirmed that a level of saturation had been achieved, with no novel findings emerging at this stage.

To investigate the influence of participants’ capabilities, opportunities and motivations on guideline implementation using the COM-B model, a deductive approach was considered to be the most appropriate for initial analysis [11]. Quotations identified as relevant to guideline implementation were considered in relation to the six COM-B sub-components (physical and psychological capability; reflective and automatic motivation; and physical and social opportunity—see Additional file 1 for definitions and examples) and assigned to one of these sub-components. Data were dual-coded independently by LA, CL and HG. The three researchers then agreed final coding to resolve discrepancies. Disagreements were resolved by SM and MK.

The data were further analysed inductively to identify themes within COM-B sub-components [12]. LA, CL and HG identified overarching themes within each COM-B component to summarise quotes representing similar underlying ideas. These themes were confirmed in discussion with SM and MK.

Data are presented by theme rather than theoretical sub-component for conceptual accessibility.

## Results

Theme saturation was achieved, and three key themes related to the three research questions were identified:

1. Role of context in implementation—budget, capacity and political influence were important influences on implementation.
2. Limitations of research evidence—the concerns expressed about guidelines included that recommendations are presented in the abstract, lack specificity and do not address complexity or local variation.
3. Using local evidence—local evidence was seen as very important, being used to provide context for recommendations and sometimes being used instead of recommendations when they conflicted with local evidence.

### Role of context in implementation

Elected members and public health team members considered specific aspects of context to be important influences on how guidelines were implemented. The limited budget of local government and accommodating political perspectives influenced how and which recommendations were implemented. Participants acknowledged that factors beyond evidence influenced implementation of recommendations.

I don’t think it’s as straightforward as to say it was all implemented, and therefore it was all evidence-based. (Site 2, Off 3)

#### Context is important

Context was identified as important in implementing recommendations but was seen to be missing from the way recommendations are framed.

And I know putting it on the web’s brilliant and I do think the pathways do help, but the only problem with the pathways is you see the recommendation of it out of context and its bias of recommendations (don’t serve who they are supposed to) (Site 4, Off 4)

Some participants challenged the traditional evidence-based model arguing that contextual factors including public preference, i.e. what the members of the public say they would like, and resources were as important as evidence in informing implementation.

We tend to talk about evidence-based decision making…Which is part of this delusory narrative… there are other things that have a place…A big bit of which, equally important, is public preference… And there are other things, in that model, such as, what resources you’ve got…Because there’s no point in the guidance being separated from capacity…So, I think, one of the things that would help in the future, is, for a bit more work to be done, on that conceptual model. (Site 1, Off 1)

The challenge for NICE is that this is not an environment where you can be driven totally by evidence or by science (Site 2, Off 1)

The world’s more complex, than the NICE evidence base, really, can do it. (Site 1, Off 1)

But participants also acknowledged that the balance of different contextual influences will vary across local governments.

In relation to any of these major public health issues, it’s a range of different things and, kind of, no one knows the exact balance because that answer will be different with different people in different communities. (Site 4, Cllr 1)

Participants conceptualised the evidence-based based model as a ‘platonic world’ where evidence drives implementation, contrasted with the ‘real world’ where other factors influence implementation.

What’s the difference between, this kind of platonic, perfect world, in which evidence-based decision making happens… And everything is rational. And, the real world, where, a whole lot of other factors, become part of the decision making process. And, what you’re trying to do is, balance, and make those as effective and rational as possible, within certain parameters…And that situational decision making, is something which we haven’t got a model for…The model is based, on here’s the evidence, everybody should do what the evidence says, because the evidence is right, and this is what it says. And, it’s kind of really, I just think, we should be a bit more sophisticated, in the traction model, or something. I can’t, it’s… I’m not quite describing it properly, but the way it gets taken up in the real world (Site 1, Off 1)

This was echoed by others acknowledging that the evidence needs to ‘speak the different language’ in local government.

This is about the evidence we use, kind of, transferring with us into local authorities, where the evidence needs to speak the different language that we need to speak (Site 1, Off 2)

#### Limited resources

Implementation of recommendations is hindered by a lack of capacity as staff time is taken up with administrative duties.

I met with the obesity team last week in the council and they said they have no time, they spend their whole time reviewing contracts and planning to get new contracts set up for 2016. So if I want to do anything in driving forward the obesity strategy, I must provide management resource from the CCG. (Site 1, Off 5)

Recent budget cuts are viewed as an opportunity by some to deliver more effective services.

For the last maybe 20, 30 years, funding has been quite stable, but then in the last five years, we’ve had to change rapidly and do things very differently, and sometimes, out of adversity, you actually come up with better solutions (Site 1, Off 5)

#### Political influences

There was the perception that implementation was more difficult in local government compared to that in NHS because political preference would take priority.

I know councils don't like being told what to do. They don’t like dicta and whereas the NHS might be able to say through its hierarchy of command and control, NICE has said this is, you know, this has passed through the hurdles, it must be implemented within three months, they can do that. You can't tell a local authority to do that. They say, well, thank you for your opinion. We'll weigh it up carefully and we'll do what our voters tell us. Welcome to democracy. (Site 3, Off 1)^1^

But this view was not shared by all.

The NHS has a very strong narrative, that it tells itself, that it’s evidence-based, and scientific, and rigorous, and everything like that. And that local government decides thing on whim, and political priorities. My experience of coming over, in to local government, was actually, that the truth of that is much more complex (Site 1, Off 1)

Some participants perceived evidence-based recommendations as being used to respond to political influence.

Well, as you know, every politician works on an anecdote. We have to use evidence either to support or refute the anecdote and sometimes you get overruled. If you manage to … ensure the evidence base is followed 75% to 85% of the time probably in this environment, we’re doing pretty well. (Site 4, Off 1)

### How useful guidelines are perceived to be

Participants highlighted limitations in the evidence used to inform recommendations—it was out of date, and in the way the evidence was presented in recommendations, they lacked specificity and did not take account of complexity and scale.

#### Evidence misses complexity and scale

Some participants appeared to think that national recommendations were of limited relevance at the local level.

I would say, is that some people have a view, that NICE guidance is not realistic, to the local situation, sometimes. (Site 2, Off 4)

However, there appeared to be two views on the way this limited relevance was manifest. Some perceived recommendations to be too broad to capture complexity. For example, where evidence informing recommendations is drawn from a number of interventions delivered as part of a strategy, there was perceived to be little guidance on which specific aspects of the package were effective.

There was a really broad recommendation and it was about doing the system change stuff, but because the evidence was based on a big system change, we know that worked, but actually what you want to understand is smaller scale stuff. What difference does this particular intervention do? And it’s quite hard to do that if, you know, it’s been implemented as part of the big package of things, and also the context is just really important. (Site 4, Off 4)

Or where recommendations were not relevant to a local population.

National evidence is important in terms of big areas, volume areas. I think where it is less effective is being able to allow you to translate that to your local population, and, depending on your population mix, something which is a big issue nationally may not necessarily translate across to your local population. So, let’s take, for example, in xxxx, you know, around 26% ethnic minority communities; that isn’t the picture nationally. So, you see, the problem that you’ve got is to meet and understand and reflect your local needs. (Site 1, Off 5)

For others, recommendations were viewed as missing the ‘big picture’.

It will give you very sophisticated analysis of, how to increase x target group, to three times 30 per week. But, it misses some of the big, systemic questions. It’s always down to a small project level, intervention level. It doesn’t look at mainstream world, changing, the way it is. (Site 1, Off 1)

To address the issues of complexity and scale, some public health teams went back to the original evidence informing the recommendations to determine the extent to which they were relevant to their local situation.

I try to get my Health Intelligence Team when they’re looking at it, to actually look at the research that was underpinning it as well, because I think sometimes if it’s based on these big studies and there are all these big, grand recommendations you think, actually that’s not always helpful locally when you’re doing some bits of it but you just want to know, will this extra thing work or not? (Site 4, Off 4)

#### Research evidence is out of date

The time lag between research evidence being produced, synthesised and informing recommendations was contrasted with the perceived speed of local government decision making.

The councillors knew exactly what was going on, how it would work, who, or how it was working, how it was evolving. It was in real time, happened in the last 12 months. The problem with NICE guidance is, it would never capture that sort of phenomenon. …NICE guidance, by definition, can only include evidence, that’s based on well-funded research, that appears in peer reviewed journals, that meets research threshold and criteria, that by definition, by the time it ends up in an effectiveness review, is three years old, at least. So, it’s always behind time, influenced by who’s funded the research, and the way they view and frame their world, and actually, their economic interest as well, in terms of their clinical guidance. So already, you’ve got a research time, and framing bias, in the NICE guidance. And, that’s very problematic, because, in, when you’re trying to solve complex problems, in real time, in a democratic setting, you need a different way of working, and a different, a different framing of solutions, and what evidence counts (Site 1, Off 1)

#### Specifying process not just outcome

Guidelines were thought to lack specificity and were considered to be framed in terms of outcome rather than behaviours involved in implementation.

I think sometimes NICE recommendations can seem a little bit vague at times, quite abstract (Site 2, Off 7)

I think sometimes the recommendations aren’t specific enough. It’s really not clear. …some of the recommendations sometimes come across as an outcome that you want to achieve, whereas I think practically quite often you want recommendations about what activity would give you that outcome. (Site 4, Off 4)

Some participants found the recommendations hard to read initially.

I remember when I went reading guidance and thinking, god this is so turgid and just like thinking, I’m just not going to read it. Now I’ve got used to it, I don’t really have a problem … just think it feels a bit like an academic report. (Site 4, Off 4)

Using case studies to illustrate implementation was suggested as a way of making recommendations less abstract.

I think if you can ground them… more sort of practical examples, case studies, things like that, but again it’s always got to be concise (Site 2, Off 7)

### Using local evidence

Local evidence was used in most cases to provide the context for recommendations. Local evidence took precedence where recommendations were viewed as not relevant to the local situation or in conflict with local evidence.

I don’t necessarily think [the local authority understanding of what evidence is] is always a different standards thing, it's just a different interpretation of how significant evidence should play in decision making. (Site 4, Off 3)

People… will dismiss it, because they feel it was done under… research conditions, and doesn’t really apply to the real world. (Site 2, Off 4)

#### Local evidence provides context for national recommendations

Participants described the process of combining recommendations and local evidence.

We’re doing something similar looking at the obesity pathway, because we’re bringing in people from… because obviously this is a system wide thing that needs to happen, and we have used guidance to come up with recommendations, but we’ve invited them to feed in. So, you know, this is what the guidance says, what does the local evidence say? So it does help us to have conversations about things, I think, and they have different types of evidence sometimes as well. (Site 4, Off 4)

We will go to what happens in [this part of] London, who else is doing something, or what happens in, I think it’s xxxx or xxxx, which borough is similar demographically to [this borough], and what’s happening in these areas. So we will either follow the national Guidance or we will look at other areas and see what best practice we can then apply to xxxx (Site 2, Off 6)

We’ve got a very diverse borough. It’s quite different demographically to the rest of xxxx, the rest of the county. I think there’s something about taking the national evidence, taking the national guidance and then always asking the question what does this mean for us. Does this speak to our community, you know? Does this take into account ethnicity? Does this take into account our levels of deprivation, etc? Does this apply in this case? And often it does. Sometimes you might think, actually, there’s shifts we need to make. It’s about localising the national. I probably do start with the national. (Site 2, Off 2)

Some viewed implementing public health recommendations as an ‘art’.

It’s how you transfer that into a local area, I think that’s the art of public health, I guess, because there are – does it – is it – is – can you apply it to the local area? And I think you would always use that as a framework to go and then audit, check what we’re doing locally, is it in line with this, that and the other? So you check all that out. And where there is a difference it’s understanding, why is there a difference? (Site 1, Off 3)

The limitations of local evidence for example, service user feedback, was acknowledged, but was still viewed as an important aspect of implementation.

So do acknowledge that any local intelligence from research or any local engagement activities or surveys or fieldwork; it’s less strong because it’s not been through that rigour of process, but valid as a piece of… for what it is in terms of, this is public opinion, service user feedback, it’s a sense of opinion or feeling around this topic of what needs to be done and what the priority areas might be to be addressed. (Site 1, Off 3)

#### Local evidence can be more persuasive than national recommendations

Both elected council members and members of the public health team cited examples where local evidence was more persuasive than national recommendations.

I think, very practical evidence of, you know, showing me a, sort of, case study, if you like, which has been done on the scale somewhere else. And so they would be probably the most persuasive types of evidence, and I think the more it’s, sort of, national or the more it’s vague or it’s less specific, then the less persuasive that would be (Site 4, Cllr 1)

If there’s a national guideline for something, we should aim to hit the target but make sure that it’s not to the detriment of what we’re providing for a person. Sometimes we miss targets because we are doing things differently. So targets are all well and good as long as they are effective. (Site 3, Cllr 2)

It might be NICE guidance, but we might say, well that won’t work in xxxx, because we know of x, y, z issues. (Site 2, Off 6)

But that sense of, again, NICE guidance, well this is what is says but it doesn't fit our local area, we didn't see any fit for us. Without necessarily looking at the detail of it. Just, it's coming from outside therefore we probably shouldn't have anything to do with it. (Site 4, Off 3).

## Discussion

### Summary of findings

The context in which recommendations are implemented, resources and political influences were perceived to influence approaches to implementation. Guidelines were thought to be useful; however, participants identified several limitations. These limitations included the following: first, recommendations not addressing the complexities, local nuances and scale faced by local government when implementing recommendations; second, a view that the research evidence on which the recommendations were based was sometimes out of date due to the time taken to produce evidence-based guidelines; and third, that recommendations focused on desired outcome rather than the process public health practitioners should undertake in order to achieve that outcome.

Limited support was found for the linear evidence-guidelines-practice model as local evidence was seen as the starting point to provide a context for implementing recommendations, or in some cases, recommendations were disregarded in favour of local evidence where they were not specific enough or where they contradicted local evidence.

### How do findings compare with other literature?

The finding that recommendations do not align with context is echoed in the global public health setting, suggesting that evidence used to inform public health policy does not take account of contextual factors such as implementation in low-income settings [13] or lack of resources. Limited capacity to deliver public health services recommended in guidelines is likely to become more of an issue of over the next few years with further planned budget cuts in local government of 4% per annum until 2020 [14].

Participants in this study reported that the lack of specificity of recommendations was a barrier to communication. Similarly, a study of implementing evidence-based recommendations in the UK National Health Service reported using examples of local initiatives were helpful where there was a lack of clarity on how recommendations should be implemented [15]. Writing about global health policymaking, Yamey and Feacham [16] noted a lack of robust evidence for what works regarding interventions to promote public health. In the context of public health in local government, behavioural science can help here by providing frameworks to guide the design and evaluation of interventions. Generating evidence for what works through robust evaluation in turn will inform the development of recommendations in evidence-based guidelines.

The emphasis on the use of local evidence over national recommendations chimes with the extant literature. An investigation into sources of evidence used by UK public health policymakers reported that experts in the area, principally directors of public health and council officers, were used as sources of evidence more often than NICE guidance [17]. The same study also noted a potential under-use of academics and researchers as sources of information for public health policymaking. Similar findings have been reported in Australia where evidence such as community perspectives are used more commonly than research evidence in informing public health policy [18].

### Implications for practice

#### Implications for producing evidence-based public health guidance

Arguably a priority for those producing evidence-based guidance for public health is also to provide more information on how to implement recommendations for public health practitioners. These could include case studies of recommendation implementation illustrating how local context can be taken into account. Participants cited examples of learning from the best practice elsewhere to guide implementation and suggested case studies of implementing recommendations be included in guidance as a way of addressing the lack of specificity.

It could be argued that public health decision-makers and practitioners in local government need to develop skills in understanding research and the practice of implementing evidence-based recommendations. However, recommendations based largely on academic research also need to be fit for purpose so that they are meaningful to decision-makers and practitioners [19] and take account of decision-makers’ and practitioners’ needs [20]. International research indicates that decision-makers in local government working in areas allied to health prioritise local relevance and case studies over scientific rigour [21]. We join others [4, 21] in arguing that it is the responsibility both of decision-makers, practitioners and those producing evidence to work together and produce relevant evidence as the basis for more readily implementable recommendations. These methods would need to start from the way that local government perceive and formulate problems and priorities within a resource-limited environment and with what they are already doing about the issues. The methods will need to be able to capture the local variations and diversity of communities and populations as well as specific local economic circumstances. The methods will also need to be able to reflect the ways that groups and institutions in local areas relate to each other, rather than focussing on individual people and their health problems or health behaviours. These data could then lead to the generation of research questions which would then drive the search for evidence—thus reversing the pipeline.

A second priority is making recommendations as specific as possible to support implementation and to present these in an accessible style and format.

Limited financial resources were identified by participants as a barrier to implementing recommendations. Some participants noted that where cost saving in guidance was mentioned, it was in relation to costs saved to the NHS. In order to make guidance more relevant to local government, a demonstration in guidance of costs saved to local government budgets may support implementation of recommendations.

#### Implications for implementing recommendations

Where evidence is limited or non-existent public health practitioners can use tools and methods developed in behavioural science to inform the design of public health interventions [22]. As noted in Oliver et al. [17], academics and researchers’ expertise could be more optimally drawn on to inform the design and implementation of interventions to promote public health.

### Limitations

As for many studies with a qualitative interview design, the main limitations of this investigation are the self-selection of a modest sample size. However, because the sample includes a range of local government employees from project officers to directors of public health as well as elected members of councils, it is unlikely these limitations compromise the integrity of the study.

## Conclusions

The evidence-based medicine model is premised on a linear pathway from primary research evidence to the appraisal and synthesis of the evidence, to the development of guidelines, and on to practice. In clinical medicine, guidelines must however be used with an absolute regard for the needs of the individual patient in front of the physician; clinical judgements must always therefore be made. In the words of Sir Michael Rawlins, the first chair of NICE, ‘Guidelines are not tramlines!’(personal communication).

In public health, there is no individual patient, so clinical judgement about individuals is not really relevant. Instead the question is whether the guideline has local applicability to local populations. Public health guidelines are based on the best national and international research evidence and will have been assessed for their internal validity. While NICE’s public health committees work hard to assess transferability and external validity, the particular needs of local communities generated by local circumstances may well confound their efforts so long as the guideline production pipeline works in the conventional direction of evidence-based medicine. This knowledge transfer problem was thrown into stark relief with the move in England of much public health from NHS to local government, where the standard assumptions of evidence-based medicine are not universally shared and where the methods of traditional guideline production are not necessarily appropriate.

Public health is characterised by much more diverse and less well-defined problems than clinical medicine and has complex pathways of action and intervention [23–26]. Pre 2013 NICE had developed its public health methods to take account of this but on the basis that the highly specific needs and priorities of particular local populations had to be left to local teams to interpret. As we have shown, with the move to local government, there was a surprising emergence of an expectation in local government that NICE national guidelines, based on the evidence, would come ready-made for local government! Because they did not, participants in this study focussed more on the limits rather than benefits of national application of guidance, namely that recommendations were ‘vague’ and not applicable in the ‘real world’.

During the planning for the reorganisation of public health into local government before 2013, there was no public discussion about the knowledge transfer issues which were going to arise as a consequence of the reforms. NICE was not consulted by the Department of Health and the learning that NICE had already accumulated about implementation in local government was not used to inform the process or the structures of engagement between the centre and local government post-2013 [5]. The research reported in this paper was funded, independently of the Department of Health and Public Health England (which in the new arrangements was supposed to be the principal conduit to local government) in order to gather primary data about what the reality of the new relationships are and how to rethink the role of guidelines in the new environment. The assumption that appears to have infused thinking of the authors of the White Paper which preceded the reforms was that it was up to local government to up their game and get used to the processes and practices of evidence-based public health. The world of public health, indeed the world of knowledge transfer does not work like that, as this research clearly shows. If guidelines are to match local needs in the ways that some participants argued for, guidelines will have to be produced very differently. This will require a fundamental rethink about the way guidelines are developed. Organisations like NICE (and Public Health England) would need to develop methods which will facilitate the re-engineering of the guideline development process in public health (and in other areas where the local government focus is the population rather than an individual).

## Appendix

### Interview schedules

#### Councillor interview schedule

Thank you for agreeing to talk to me today. The purpose of this project is to find out about how local government are currently using evidence and guidance in public health work, and how NICE guidance can best support local government work.

By ‘public health’, we mean any of the work carried out by local government that aims to improve public health, for example by enabling people to access information, advice or services about health issues and intervening to promote behaviours that increase health and discourage behaviours that reduce it.

With the recent transition of public health responsibilities from the NHS to local government, we realise that many people will not be familiar with NICE guidance and may not find it useful, or would like to use it but are not sure how. In order to make this guidance as useful as possible, we need to understand the extent to which people in different roles within local government are familiar with the guidance, and use it.

**Table:**

	Interview question		
1	Can you tell me a little bit about your current role at the Council, and how long you’ve been in this role?		
2	(If not clear from previous answer) What involvement do you currently have in issues relating to public health at the Council?		
3	Prior to your current role, has your background included any work in health-related fields (through employment, voluntarily or through local politics)?		
4	Responsibility for many public health functions transferred from the NHS to local government in 2013. What changes have you noticed at the Council since then with respect to public health? What about social care?		
5	In your experience, how does the Council decide the priority issues around public health to focus on?		
6	When policy or action around public health issues are developed by the Council, what role do Councillors play and what role do officers play? How do you interact?		
7	What kinds of evidence or guidelines do the Council refer to when developing policy or action around public health issues? (probe for local and national types of evidence)		
8	Do you personally have the opportunity to look at evidence or guidelines when considering public health issues?		
9	What do you think the strengths and limitations of local and national evidence are?		
10	What do you think of as weak or strong evidence?		
11	And where on that spectrum of weak to strong evidence does the type of evidence you mostly use sit?		
12	In your experience, how does the political side of the Council’s role interact with the use of evidence or guidelines in public health work?		
13	NICE produces guidelines for people working on public health issues, including guidelines for local government to help them implement policies that address public health priorities. Is this NICE guidance something you personally are aware of?		
14	If yes	How did you come to hear of the NICE guidance?	
15		Are you aware of the NICE guidance on smoking, healthy eating, physical activity, behaviour change or engaging communities? (Ask about each slowly)	
16		Have you read any of the NICE guidance itself?	
17		How did you access the NICE guidance?	
18		Were you able to apply the NICE guidance in your role as a councillor?	
19		If yes	How did you apply the guidance?
20			Did you look at the evidence supporting the guidance? If yes, how useful was this, and why?
21			What impact do you think using the NICE guidance had on that piece of work?
22			What factors do you think helped you in your role as a Councillor to apply the guidance?
23		If no	Why was this?
24		Thinking about the role of Councillors in particular, what barriers do you think there are to using NICE guidance?	
25		And thinking about the Council more generally, what barriers do you think there are to the use of NICE guidance?	
26		Thinking about the role of Councillors in particular, is there anything that you think could have helped you to apply the NICE guidance?	
27		And thinking about the Council more generally, what do you think could help the Council to apply the NICE guidance?	
28		How relevant or useful is the NICE guidance to you as a Councillor?	
29		How could NICE guidance be made more accessible and useable by you?	
30	Are you familiar with the public health and local government briefings produced by NICE?		
31	Are you familiar with the pathways NICE have developed for accessing this information?		
32	NICE also develops quality standards alongside its guidance. Are you aware of NICE quality standards? If yes, Which ones have you heard about?		
33	Are you aware of the Public Health Outcomes Framework?		
34	Now that you’ve heard the questions and the things that we’re interested in, is there anything further you’d like to add, or any other area that you think might be important that we haven’t mentioned?		

#### Public health officers (DPH, PH team etc) interview schedule

Thank you for agreeing to talk to me today. The purpose of this project is to find out about how local government are currently using evidence and guidance in public health work, and how NICE guidance can best support local government work.

By ‘public health’, we mean any of the work carried out by local government that aims to improve public health, for example by enabling people to access information, advice or services about health issues and intervening to promote behaviours that increase health and discourage behaviours that reduce it.

With the recent transition of public health responsibilities from the NHS to local government, we realise that many people will not be familiar with NICE guidance, or would like to use it but are not sure how. In order to make this guidance as useful as possible, we need to understand the extent to which people in different roles within local government are familiar with the guidance, and use it.

**Table:**

	Interview question		
1	Can you tell me a little bit about your current role at the Council?		
2	(If not in obvious public health role) How does your role relate to promoting public health?		
3	(If not previously answered) What involvement do you have in implementing public health policy or strategies or interventions at the Council?		
4	Responsibility for public health transferred from the NHS to local government in 2013. Were you already working for the Council when this happened, or did you transfer from the NHS?		
5	What changes did you notice after April 2013 with respect to public health? What about social care?		
6	(If not obvious from job role) How close are you personally to decision making about policy and action in your department?		
7	In your experience, how does the Council decide the priority issues around public health to focus on?		
8	What kinds of evidence or guidance do the Council use when deciding what to do about these public health issues? (probe for local and national types of evidence)		
9	What do you think the strengths and limitations of local and national evidence are?		
10	What do you think of as weak or strong evidence?		
11	And where on that spectrum of weak to strong evidence does the type of evidence you mostly use sit?		
12	Are there systems in place for selecting the guidance or evidence used to support decision-making?		
13	Are there systems in place to record or track the use of guidance or evidence in decision-making?		
14	In your experience, how does the political side of the Council’s role interact with the use of evidence or guidelines in public health work?		
15	In your experience, when different council departments are working together on public health-related issues, how is evidence or guidance used?		
16	In your experience, how is evidence or guidance used when the council works in partnership with other organisations?		
17	Are there systems in place for monitoring how well the aims of public health strategies, plans and interventions are met?		
18	NICE produces guidelines for people working on public health issues, including guidelines for local government to help them implement policies that address public health priorities. Is this NICE guidance something you personally are aware of?		
19	If yes	How did you come to hear of the NICE guidance?	
20		Are you aware of the NICE guidance on smoking, healthy eating, physical activity, behaviour change or engaging communities? (Ask about each in turn)	
21		Have you read any of the NICE guidance itself?	
22		How did you access the NICE guidance?	
23		Were you able to apply the NICE guidance to your work?	
24		If yes	How did you apply the guidance?
25			Did you look at the evidence supporting the guidance? If yes, how useful was this, and why?
26			What impact do you think using the NICE guidance had on that piece of work?
27			How could that impact be measured?
28			What factors do you think helped you to apply the guidance?
29		If no	Why was this?
30		What barriers do you think there are to applying the NICE guidance?	
31		Is there anything that you think could have helped you/your Council to apply the NICE guidance?	
32		How relevant or useful is the NICE guidance to you in your current role?	
33		If relevant, how could NICE guidance be made more accessible and useable by you?	
34	Are you familiar with the public health and local government briefings produced by NICE? (prompt for details if yes)		
35	Are you familiar with the pathways NICE have developed for accessing this information? (prompt for details if yes)		
36	NICE also develops quality standards alongside its guidance. Are you aware of NICE quality standards? If yes, Which ones have you heard about?		
37	Are you aware of the Public Health Outcomes Framework?		
38	If yes		Does the Council use it or refer to it? If yes, how?
39	Now that you’ve heard the questions and the things that we’re interested in, is there anything further you’d like to add, or any other area that you think might be important that we haven’t mentioned?		

## Additional file

Additional file 1: Definitions and examples of the COM-B model sub-components. (DOCX 12 kb)

## Footnotes

1 This quotation is referring to NICE Technology Appraisals and not public health recommendations. There was no imperative to comply with NICE public health guidelines in the pre-2013 NHS primary care system.
