# Supplementary material for: Reversing the pipeline? Implementing public health evidence-based guidance in english local government
Source: Implement Sci. 2017 May 12;12:63. doi: 10.1186/s13012-017-0589-5 (PMC5429536; doi:10.1186/s13012-017-0589-5)
Supplement: Additional file 1: — Definitions and examples of the COM-B model sub-components. (DOCX 12 kb) [file 13012_2017_589_MOESM1_ESM.docx]

**Additional file 1: Definitions and examples of the COM-B model sub-components**

| **COM-B model component**  Definition | ***Example*** |
| --- | --- |
| **Physical capability**  Physical skill, strength or stamina | *Having the skill to take a blood sample* |
| **Psychological capability**  Knowledge or psychological skills, strength or stamina to engage in the necessary thought processes | *Understanding the impact of CO2 on the environment* |
| **Physical opportunity**  Opportunity afforded by the environment involving time, resources, locations, physical barriers | *Being able to go running because one owns appropriate shoes* |
| **Social opportunity**  Opportunity afforded by interpersonal influences, social cues and cultural norms that influence the way that we think about things, e.g. the words and concepts that make up our language | *Being able to smoke in the house of someone who smokes but not in the middle of a boardroom meeting* |
| **Reflective motivation**  Reflective processes involving plans and evaluations | *Intending to stop smoking* |
| **Automatic motivation**  Automatic processes involving emotional reactions, impulses and reflex responses that arise from associative learning and/or innate dispositions | *Feeling anticipated pleasure at the prospect of eating a piece of chocolate cake* |
